# Supplementary figures and images for: Beta-Hydroxyisovaleryl-Shikonin Eradicates Epithelial Cell Adhesion Molecule-Positive Liver Cancer Stem Cells by Suppressing dUTP Pyrophosphatase Expression
Source: Int J Mol Sci. 2023 Nov 14;24(22):16283. doi: 10.3390/ijms242216283 (PMC10671815; doi:10.3390/ijms242216283)

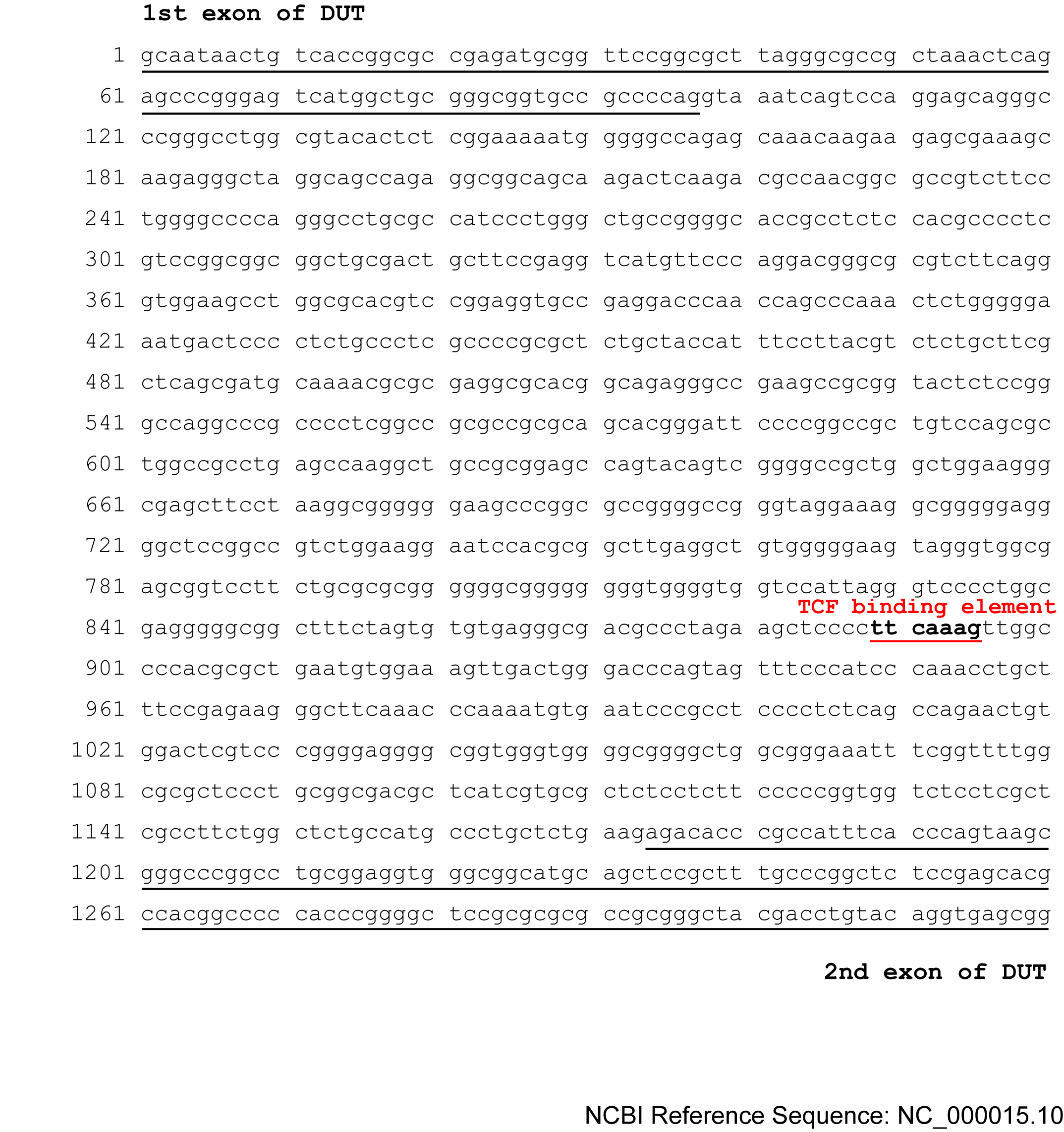

Supplement: Supplementary file 1 [file ijms-24-16283-s001.zip › ijms-2692829-supplementary Figure S1.tif]
